# Gut Microbiota and Coronary Artery Disease: Current Therapeutic Perspectives

**DOI:** 10.3390/metabo13020256

**Published:** 2023-02-09

**Authors:** Themistoklis Katsimichas, Panagiotis Theofilis, Konstantinos Tsioufis, Dimitris Tousoulis

**Affiliations:** 1st Cardiology Department, National and Kapodistrian University of Athens, Vas. Sofias Avenue 114, 11527 Athens, Greece

**Keywords:** gut microbiota, TMAO, probiotics, prebiotics, synbiotics, coronary artery disease, TMAO inhibitors

## Abstract

The human gut microbiota is the community of microorganisms living in the human gut. This microbial ecosystem contains bacteria beneficial to their host and plays important roles in human physiology, participating in energy harvest from indigestible fiber, vitamin synthesis, and regulation of the immune system, among others. Accumulating evidence suggests a possible link between compositional and metabolic aberrations of the gut microbiota and coronary artery disease in humans. Manipulating the gut microbiota through targeted interventions is an emerging field of science, aiming at reducing the risk of disease. Among the interventions with the most promising results are probiotics, prebiotics, synbiotics, and trimethylamine N-oxide (TMAO) inhibitors. Contemporary studies of probiotics have shown an improvement of inflammation and endothelial cell function, paired with attenuated extracellular matrix remodeling and TMAO production. *Lactobacilli*, *Bifidobacteria*, and *Bacteroides* are some of the most well studied probiotics in experimental and clinical settings. Prebiotics may also decrease inflammation and lead to reductions in blood pressure, body weight, and hyperlipidemia. Synbiotics have been associated with an improvement in glucose homeostasis and lipid abnormalities. On the contrary, no evidence yet exists on the possible benefits of postbiotic use, while the use of antibiotics is not warranted, due to potentially deleterious effects. TMAO inhibitors such as 3,3-dimethyl-1-butanol, iodomethylcholine, and fluoromethylcholine, despite still being investigated experimentally, appear to possess anti-inflammatory, antioxidant, and anti-fibrotic properties. Finally, fecal transplantation carries conflicting evidence, mandating the need for further research. In the present review we summarize the links between the gut microbiota and coronary artery disease and elaborate on the varied therapeutic measures that are being explored in this context.

## 1. Introduction

The human gut microbiota is the microbial community residing in the human gut. It comprises around 50 trillion microorganisms [1], a number roughly equal to the number of human cells. These organisms are overwhelmingly anaerobic bacteria, averaging a few hundred species per host [2]. Humans are generally colonized at birth, with a microbiota that increasingly diversifies until it reaches an adult configuration around age 3, remaining relatively stable until old age [2,3,4,5,6]. The richness and diversity of the gut microbiota is determined by numerous factors, such as geography, host genetics, and age, but primarily by host dietary habits [7,8,9]. Although not all gut bacteria are beneficial to their host, several species of mutualistic microorganisms are not only tolerated by the human immune system, but constitute metabolic participants to various host-related physiological functions. Among others, such functions include vitamin synthesis, regulation of the immune system, and the production of energy from sources which cannot be digested in the human intestinal tract [4,5]. Of particular importance is the production by the microbiota of the short-chain fatty acids (SCFA) propionate, acetate, and butyrate. Butyrate has a strong anti-inflammatory potential and constitutes the main energy source for gut epithelial cells, while SCFA as a whole may be partly responsible for the benefits derived from consumption of the fiber-rich Mediterranean diet [10,11].

Despite the fact that modern microbiota research is relatively young, mounting evidence already suggests links of microbial community compositional and/or functional aberrations to human disease, including coronary artery disease (CAD) [12,13,14]. Such aberrations (relative to control groups of subjects) are generally termed “dysbiosis”, although the lack of consensus on what is considered a normal gut microbiota makes the application of this term somewhat arbitrary. In the following sections, we will briefly summarize the links between gut bacterial dysbiosis and CAD, and focus on possible therapeutic interventions which might reduce the associated risk.

## 2. The Association of Gut Bacterial Dysbiosis with CAD

**Figure 1:**
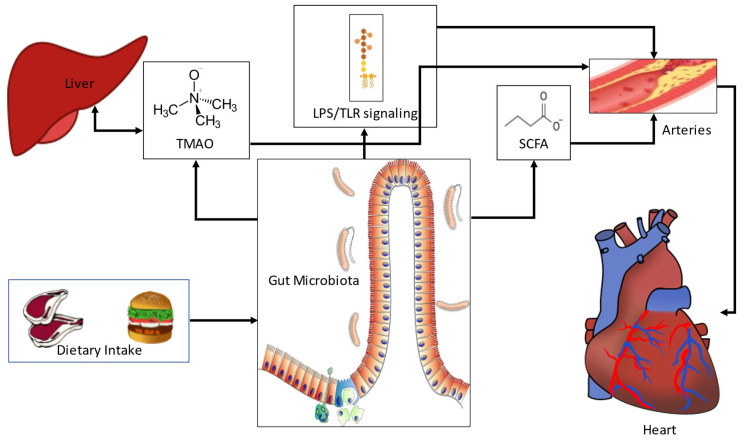
The pathophysiology of gut microbial associations with coronary artery disease. Diet critically shapes the gut microbiota, providing substrates for fermentation. Undigested fiber consumed by bacteria leads to the production of SCFA. Low levels of SCFA have been linked to host inflammation, which may influence atherosclerosis. Many bacterial species also produce TMA using choline as a source. TMA is metabolized to TMAO in the liver by flavin monooxygenases, and is associated with atherosclerosis. Bacterial LPS leaking into the circulation from the gut initiates TLR-mediated systemic inflammation, also linked to atherosclerosis. LPS: Lipopolysaccharide, SCFA: short-chain fatty acids, TLR: toll-like receptor, TMAO: trimethylamine N-oxide.

The pathophysiological basis of the association between bacterial dysbiosis and CAD may involve multiple cellular and molecular pathways that mainly relate to infection, changes in host bile acid and lipid metabolic profiles, leakage of harmful endotoxins through the gut epithelial barrier, and generation of potentially atherogenic bacterial metabolites (Figure 1). 

Infection causes systemic inflammation, which may destabilize atherosclerotic plaques. This is particularly true for respiratory pathogens [15], but it is less clear whether gut infections also lead to similar results [16]. Atherosclerotic plaques contain bacteria which may play some role in promoting atherosclerosis and could have translocated from the gut, although evidence is scarce [12]. 

Bile acid metabolism involves bacteria-mediated deconjugation and formation of secondary products, as well as bacterial influence on the bile acid receptor (farnesoid X receptor, FXR). FXR-deficient mice develop hypercholesterolemia [17], although evidence in humans is limited. 

Gut bacterial functions also affect lipid metabolism [18], but their role on the key low-density lipoprotein (LDL) molecule is obscure at best. Host-microbiota crosstalk concerning lipid metabolism is at least in part mediated by nuclear peroxisome proliferator-activated receptors (PPAR), with species-specific interactions [19]. As an example, PPARγ responds to butyrate production by gut bacteria and facilitates β-oxidation, while suppressing Nitric Oxide (NO) synthesis, maintaining the anaerobic milieu in the colonic environment and preventing dysbiosis [19,20,21]. Further, a high fat diet has been shown to procure spatial and compositional alterations of the gut microbiota, together with dysregulation of PPARγ signaling, which seems to control the spatial distribution of bacteria in the ileum of mice [22]. These phenomena were reversed by administration of a PPARγ agonist.

Gram negative bacterial wall component lipopolysaccharide (LPS), an endotoxin, has strong connections to low grade systemic inflammation in humans and its leakage into the bloodstream through a compromised or even intact epithelial barrier may contribute to atherosclerosis [23,24,25]. LPS initiates Toll-like receptor (TLR) signaling and influences circulating cytokine levels, including Tumor Necrosis Factor (TNF)—α.

Finally, certain bacterial metabolites have received attention for their potentially proatherogenic properties. Trimethylamine (TMA), a bacterial derivative of choline, is metabolized to trimethylamine N-oxide (TMAO) by flavin-containing monooxygenases (FMO) in the liver and is arguably the single most researched bacterial metabolite in relation to CAD. It was originally associated with promotion of atherosclerosis in rodents [26], and has since been extensively studied, with conflicting results. Increased TMAO levels have also been detected in non-alcoholic fatty liver disease [27], which is a known risk factor for CAD [28]. Bacterial species known to produce TMA include *Escherichia coli*, *Clostridium XIVa* strains, and *Eubacterium* species strain AB3007 [29], as well as *Anaerococcus hydrogenalis*, *Escherichia fergusonii*, *Proteus penneri*, *Providencia rettgeri*, and *Edwardsiella tarda* [30]. Many more are expected to exist, to the point that TMA production can be considered functionally redundant in the mammalian gut [31]. An additional metabolite is tryptophan; a reduced capacity of bacterial tryptophan metabolism has been linked to host metabolic dysregulation [32].

Based on the pathophysiological rationale, numerous clinical studies have been conducted in humans, mostly of an observational design (summarized in a recent position paper of the European Society of Cardiology Working Group on coronary pathophysiology and microcirculation) [33]. Multiple studies have found altered microbial diversity in subjects with atherosclerosis or atherosclerotic-related cardiovascular events. Although results are often not corroborated between studies, perhaps due to technical issues or patient background differences, an overall shift in ecological metrics such as alpha and beta diversity has been noted and individual microbial taxa differing in relative abundance have been pinpointed. As examples, stroke patients had a greater abundance of opportunistic pathogens *Enterobacter*, *Megasphaera*, *Oscillibacter*, and *Desulfovibrio*, and a smaller abundance of generally beneficial genera, such as *Bacteroides*, *Prevotella*, and *Faecalibacterium* [34]. Similarly, shotgun sequencing of the gut microbiome in a large cohort revealed a reduction in the *Bacteroides* and *Prevotella* genera, and an enrichment in the *Streptococcus* and *Escherichia* genera in atherosclerotic patients [35]. Patients in this cohort also had differences in other bacterial species and strains, such as more *Klebsiella* spp. and *Enterobacter aerogenes,* and fewer butyrate-producing bacteria, including *Roseburia intestinalis* and *Faecalibacterium* cf. *prausnitzii*. 

More importantly, metabolic characteristics of the gut microbiota of atherosclerotic patients were shown to differ in comparison to healthy control subjects, pointing towards a larger inflammatory effect [35]. In yet another large cohort study, *Enterococcus* was significantly enriched, while *Faecalibacterium*, *Subdoligranulum*, *Roseburia*, and *Eubacterium rectale* were significantly depleted in CAD patients [36]. This study also verified metabolic shifts in CAD related bacterial communities, including enhanced LPS biosynthesis and increased protein and tryptophan metabolism. Lastly, TMAO has been linked to an increased cardiovascular risk in many studies and meta-analyses [37,38,39], although conflicting results exist and causality has not been proven in humans [40,41].

It is not apparent why differences exist between healthy subjects and CAD patients. The observational nature of the vast majority of the relative studies precludes any inference of causality and since a single standardized research protocol is generally not implemented internationally, comparison between studies is challenging at best.

**Figure 2:**
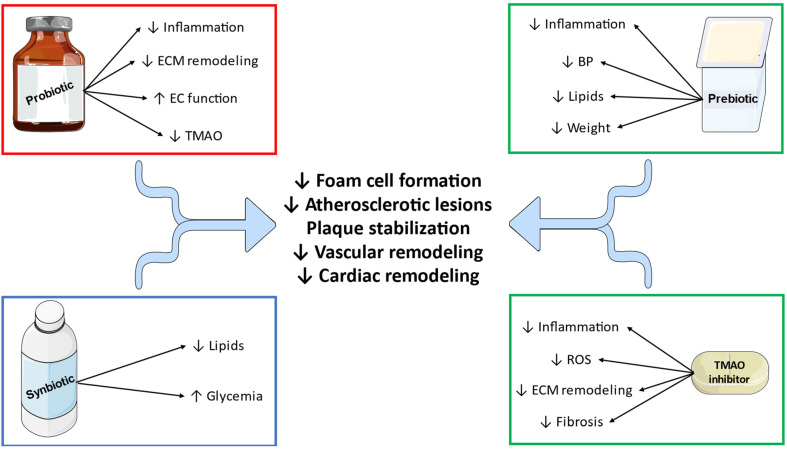
Beneficial effects of gut microbiota modulators in coronary artery disease. ECM: extracellular matrix, EC: endothelial cell, TMAO: trimethylamine N-oxide, BP: blood pressure, ROS: reactive oxygen species. ↓: decreased, ↑ increased.

Conceivably, the links between gut bacterial dysbiosis and CAD have generated interest in therapeutic interventions that may modulate the possible risk (Table 1 and Figure 2). The following section will focus on the relative research efforts.

## 3. Current Therapeutic Interventions

### 3.1. Probiotics

Probiotics are ‘live microorganisms that, when administered in adequate amounts, confer a health benefit on the host’ [58]. The most common probiotics are *Lactobacilli* and *Bifidobacteria*. Many preparations are commercially available and are mostly used as dietary supplements. Administration of probiotics may confer beneficial cardiovascular actions, according to findings from several mechanistic studies. More specifically, reports of anti-inflammatory, antioxidant, antithrombotic, and endothelium-protective effects have been published [59]. Moreover, probiotics could restore and strengthen the intestinal barrier integrity, limit hazardous LPS leakage, and suppress TMAO formation [59]. They may also promote bile acid deconjugation, thus increasing bile acid excretion and cholesterol utilization [33]. Hence, they may possess anti-atherosclerotic potential. A previous review of their potentially anti-atherosclerotic effects can be found elsewhere [60].

*Lactobacilli* are among the most well-studied probiotics with regards to their cardiovascular benefit. In a mechanistic study in a swine model of CAD, an anti-inflammatory and antioxidant effect mediated by the induction of NF-E2-related factor 2 in the ischemic myocardium could have been the outcome of *Lactobacillus plantarum* supplementation [61]. This has been further tested in men with CAD, with *Lactobacillus plantarum* administration resulting in anti-inflammatory and endothelium-protective effects, without affecting metabolic parameters or TMAO concentration, however [42,62]. Further, in a recently reported open-label, randomized trial of 77 dyslipidemic patients, administration of *Lactobacillus plantarum* together with a lipid-lowering agent (simvastatin 20 mg) resulted in significant improvement of the lipid profile and reduction of the calculated cardiovascular risk, compared to simvastatin 20 mg alone [63]. Concerning *Lactobacillus rhamnosus*, a randomized, double-blind trial of 44 CAD patients on a 12-week supplementation with this probiotic, together with caloric restriction, showed anti-inflammatory effects and significant weight loss, compared to caloric restriction alone [43]. Other *Lactobacilli*, such as *Lactobacillus paracasei* DTA81, have also been tested in experimental atherosclerotic conditions, with initial reports indicating an anti-inflammatory effect together with improved lipid and glucose homeostasis [64]. *Lactobacillus reuteri* administration in LDLr^−/−^ mice has also produced anti-inflammatory actions that attenuated cardiac ischemia/reperfusion injury, evidenced by reduced infarct size, without affecting cholesterol levels [44]. Oral administration of *Lactobacillus rhamnosus* GR-1 ameliorated cardiac remodelling and pump failure in rats with induced acute myocardial infarction [65]. A recent meta-analysis of studies found that *Lactobacillus acidophilus* may have a greater effect in lowering cholesterol than other probiotics [66].

*Bifidobacteria* also yield probiotic strains with potential benefit in atherosclerosis. Patients with CAD were enrolled in a randomized, double-blind, placebo-controlled trial examining the efficacy of the probiotic strain *Bifidobacterium lactis* Probio-M8 in combination with conventional treatment involving lipid-lowering (atorvastatin) and β-blockade (metoprolol) [45]. Based on the study results, the study group reported significant improvement in anginal, anxiety, and depressive symptoms assessed through the Seattle Angina Questionnaire, Self-Rating Anxiety Scale, and the Self-Rating Depression Scale, respectively, compared to the control group. Moreover, the investigators documented a greater reduction in interleukin-6 and LDL with the probiotic strain at 6 months, compared to placebo. Regarding gut microbial composition, an abundance of *Bifidobacterium adolescentis*, *Bifidobacterium animalis*, *Bifidobacterium bifidum*, and *Butyricicoccus porcorum*, together with decreased *Flavonifractor plautii* and *Parabacteroides johnsonii* was detected in probiotic-treated subjects. Interestingly, the study group participants exhibited an increase in bioactive microbial metabolites and a decrease in TMAO and proatherogenic amino acids (l-leucine, l-valine). This modulation of gut microbiota and gut metabolome profile through the probiotic strain might have been responsible for the improvement in quality of life, as well as for the reported anti-inflammatory and hypolipidemic effects.

Less evidence is available for other probiotic strains, such as *Limosilactobacillus fermentum*. Administration of a probiotic formulation containing *Liminosilactobacillus fermentum* in Wistar rats fed with a high fat diet restored gut microbial composition, which was further accompanied by attenuated metabolic and blood pressure abnormalities [67]. Moreover, the same formulation promoted anti-inflammatory and antioxidant actions at the level of the heart in female Wistar rats consuming a high fat diet [68]. Probiotics *Bacteroides vulgatus* and *Bacteroides dorei* inhibited atherosclerotic plaque formation in ApoE-deficient mice [46].

Some of the above-mentioned probiotics have also been administered in combinations. Preclinical studies have provided important insight in this regard. Male LDLr^−/−^ mice consuming a high fat diet were treated with or without a combination of probiotics (*Lactobacillus acidophilus*, *Bifidobacterium bifidum*, *Bifidobacterium animalis* subsp. *lactis*, *Lactobacillus plantarum*) for 12 weeks [47]. The investigators noted a significant reduction in the degree of aortic root occlusion paired with features of plaque stabilization (decreased plaque lipid content and macrophages, increase in α-smooth muscle cell actin) in mice receiving the probiotic combination. Moreover, a downregulated expression of several genes was seen in the study group, involving critical pathways (inflammation, lipid transport and metabolism, cell adhesion, extracellular matrix remodeling, apoptosis). Additionally, the probiotic formulation resulted in attenuated monocyte chemoattractant protein-1-induced monocyte migration, monocyte and macrophage proliferation, foam cell formation, and smooth muscle cell proliferation and migration. Furthermore, in an experimental study involving male Wistar rats fed with a high fat diet, treatment with *Lactobacillus rhamnosus* FM9 and *Limosilactobacillus fermentum* Y57 had similar efficacy in increasing high-density lipoprotein cholesterol levels compared to a lipid-lowering agent [69]. At the same time, the researchers documented a greater reduction in total cholesterol and LDL with the above-mentioned probiotics. In a human clinical study of young and middle-aged women with arterial hypertension, 8-week treatment with the combination of *Lactobacillus paracasei* LPC-37, *Lactobacillus rhamnosus HN001*, *Lactobacillus acidophilus* NCFM, and *Bifidobacterium lactis* HN019 significantly reduced fasting glucose and improved the lipid profile and autonomic modulation [70]. At the same time, a trend towards a significant blood pressure-lowering effect (systolic blood pressure reduction ~5 mmHg, diastolic blood pressure reduction ~2 mmHg) was observed. 

Although many of the above studies are of promising potential, it should be stressed that most probiotic health claims generally rely on poor evidence. The ideal probiotic strain(s) and/or dose in the context of CAD are still unspecified.

### 3.2. Prebiotics

**Figure 3:**
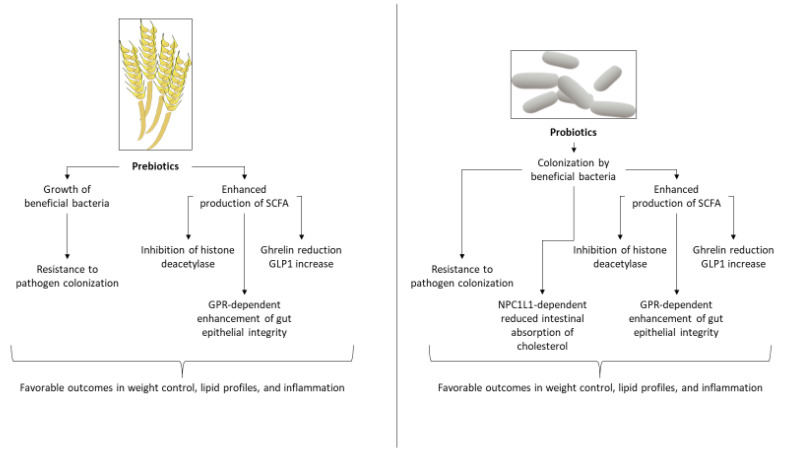
Examples of cellular and molecular mechanisms underlying the potential effects of pre- and probiotic administration in metabolic and coronary artery disease. GLP1: glucagon-like peptide-1, GPR: G-protein coupled receptor, NPC1L1: Niemann-Pick C1-like 1 protein, SCFA: short-chain fatty acids.

Prebiotics are indigestible food substances that are selectively utilized by and can promote the growth of beneficial gut bacteria, conferring a health benefit on the host [71]. Some of the most common prebiotics that have been tested in the context of cardiovascular disease (CVD) are fructooligosaccharides, inulin, galactooligosaccharides (galactans), beta glucan, Minolest, pectin polysaccharides, and chitosan oligosaccharides. Evidence from human trials suggest that they might be of value in CAD. Possible underlying mechanisms include increased production of SCFA, enhancement of gut epithelial tight junctions, and enhanced growth of beneficial bacteria [72]. More specifically, prebiotics may favor growth and metabolic functions of known beneficial bacterial genera, such as those of *Lactobacillus* and *Bifidobacterium*, some of which are themselves used as probiotics, as described above, and may antagonize growth of or colonization by pathogenic species [73]. Production of SCFA by such bacteria may promote gut epithelial integrity through G-protein coupled receptor 43 (GPR43) signaling [74]. Further, SCFA-dependent inhibition of histone deacetylase results in a robust anti-inflammatory gut environment [75,76]. Prebiotic administration may also lead to a reduction of ghrelin release and an increase of glucagon-like peptide-1 (GLP-1) production [51], favorably affecting weight control and fat accumulation (Figure 3).

However, it must be stressed that the European Food and Safety Authority generally refutes any health claim related to prebiotic preparations [77].

There are very few clinical studies directly addressing the impact of prebiotics on human CAD, although evidence exists in animal models [72,78]. One study in humans showed that treatment with beta glycans prevented ischemia/reperfusion injury in patients who underwent coronary artery bypass grafting [48]. The administration of chitosan oligosaccharides in another human trial of CAD patients improved lipid profiles, and increased the abundance of the genera *Faecalibacterium*, *Alistipes*, *Escherichia*, *Lactobacillus*, *Lactococcus*, and *Phascolarctobacterium* [79].

Generally prebiotic-specific and influenced by host dietary background, results from both animal and human studies are promising in high CAD risk disorders, such as obesity and the metabolic syndrome (for a comprehensive review see Santos-Marcos et al. [80]), as well as in healthy subjects [80]. As an example, fructooligosaccharides significantly reduce body weight and inflammatory cytokines in obese rats [49]. A reduction of inflammatory cytokines and an improvement in lipid profiles has also been reported in a randomized controlled trial of human diabetic patients taking inulin vs. placebo [50]. Also in humans, oligofructose consumption was associated with a decrease in ghrelin and weight loss, evidence that again come from a randomized controlled trial [51]. Inulin use in a human randomized control trial led to an increase of *Bifidobacterium* [81], a result that was also seen with the use of galactooligosaccharides in another randomized controlled trial that recruited obese, prediabetic humans [82]. Moreover, the use of agave fructans has been shown to increase both *Bifidobacteria* and *Lactobacilli* in yet another randomized controlled trial [83]. Further, administration of guar gum led to increases in *Bifidobacterium*, the *Clostridium coccoides* group, the *Roseburia*/*Eubacterium rectale* group, *Eubacterium hallii*, and butyrate-producing bacterium strain SS2/1, generally enhancing butyrate production in a study of healthy human subjects [84].

### 3.3. Synbiotics

Synbiotics are ‘a mixture comprising live microorganisms and substrate(s) selectively utilized by host microorganisms that confers a health benefit on the host’ [85]. Synbiotics have shown promise as interventions in human CAD and CAD related disorders, such as obesity and diabetes mellitus (reviewed in Saez-Lara et al.) [86]. As expected, the molecular mechanisms underlying the beneficial effect are those concerned with the pre- and probiotic components of the mixture, as described above. In a randomized controlled trial of diabetic patients with CAD, a synbiotic mixture of *Lactobacillus acidophilus*, *Lactobacillus casei*, *Bifidobacterium bifidum* and inulin led to improvements in glycemia and HDL [52], while another randomized controlled trial showed improvements in lipid levels in patients with type 2 diabetes consuming a mixture of *Lactobacillus sporogenes* and inulin [87]. A meta-analysis of randomized controlled trials found that synbiotic supplementation in patients with metabolic syndrome significantly reduced serum insulin levels, triglycerides, total cholesterol, LDL, waist circumference, body weight, systolic blood pressure, and serum interleukin-6, and increased HDL [88]. The most effective synbiotic in CAD is currently unknown.

### 3.4. Antibiotics

The use of antibiotics targeting atherosclerotic plaque microorganisms in CAD has failed to show any benefit and has recently been proven harmful to humans [89,90]. This approach is also harmful to scores of beneficial gut bacteria and should be abandoned.

Microbial investigation of atherosclerotic plaques has shown the existence of a diverse community of more than 50 bacterial species in coronary lesions, including *Staphylococcus* species, *Proteus vulgaris*, *Klebsiella pneumoniae*, *Chlamydia pneumoniae*, *Chlamydia trachomatis*, and *Streptococcus* species [91]. Most of these bacteria probably originate from the oral and gut flora, and translocate to the coronary arteries through bloodstream circulation [91]. 

An intriguing hypothesis had been formulated, that bacteria may at least influence the progression of atherosclerosis by induction of inflammation in the atherosclerotic plaque. One of the earliest attempts to address the possible association of a microorganism with CAD showed that increased antibodies against *Chlamydia pneumoniae* predicted cardiovascular events in patients with myocardial infarction, and that administration of azithromycin reduced this risk [92]. However, subsequent and considerably larger studies failed to show any benefit with azithromycin or gatifloxacin in the secondary prevention of events [93,94]. Finally, a comprehensive Cochrane meta-analysis involving more than 26,000 patients receiving macrolides or quinolones conclusively showed that antibiotic use has no benefit in secondary prevention and that it also has a strong trend for being harmful to humans, tending to increase all-cause mortality [89]. 

### 3.5. Postbiotics

Postbiotics have been recently defined as ‘preparations of inanimate microorganisms and/or their components that confer a health benefit on the host’ [95]. Using this definition, there is currently no evidence of any postbiotic associated effect on human CAD patients [95]. Purified bacterial metabolites that have shown effects in various health conditions do not fall under the term “postbiotic” [95]. However, components of bacterial cell walls do fall under this term and some evidence suggests a beneficial metabolic effect of purified *Akkermansia muciniphila* membrane protein Amuc_1100 in diabetic mice, implying a possible antiatherogenic role [96].

### 3.6. Additional Interventions

#### 3.6.1. TMAO Inhibition

**Figure 4:**
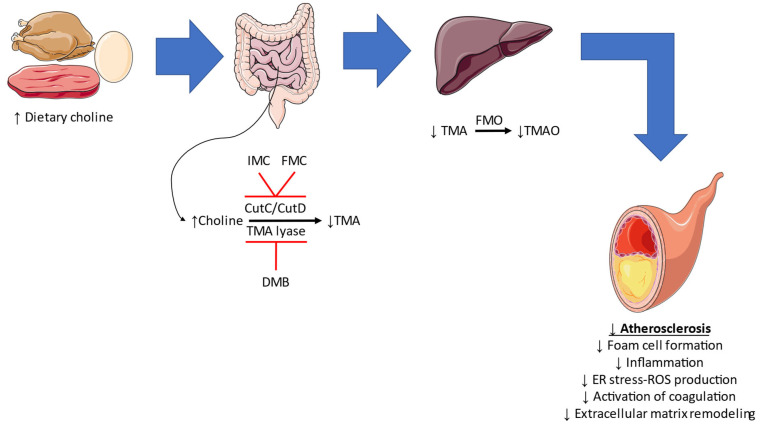
The effect of TMAO inhibition in atherosclerosis. Consumption of a high-choline diet leads to its conversion to trimethylamine (TMA) in the gut, with the aid of CutC/CutD and TMA-lyase. Inhibition of these enzymes by iodomethylcholine (IMC)/fluoromethylcholine (FMC) and 3,3-dimethyl-1-butanol (DMB) leads to lower trimethylamine N-Oxide (TMAO) production in the liver. As a result, atherosclerosis may be attenuated through multiple mechanisms. FMO: flavin-containing monooxygenase, ER: endoplasmic reticulum, ROS: reactive oxygen species.

As mentioned, TMAO has garnered significant scientific attention and studies aiming to inhibit its production and investigate the result have been conducted. In the earliest study, by Wang et al., a structural analog of choline, 3,3-dimethyl-1-butanol (DMB), inhibited TMA-lyase activity, decrease TMA levels, and ultimately lowered TMAO levels in mice under a high-choline or L-carnitine diet [53]. Interestingly, this was accompanied by reduced foam cell formation and diminished atherosclerotic lesion development in ApoE^−/−^ mice. In another study, DMB ameliorated cardiac dysfunction in male CD1 mice fed with a high fat diet, without inducing alterations in body weight or cholesterol levels [97]. The cardioprotective effect could be attributed to the anti-inflammatory and anti-fibrotic effects observed histologically. Such observations were reported in a rat model of type 2 cardiorenal syndrome [98]. Anti-inflammatory, antioxidant, and endothelial protective actions were further noted in aged Fischer-344 rats and aged C57BL/6N treated with DMB [99,100]. In a recently reported study, DMB was administered in a wild-type mouse model of partial carotid artery ligation together with a high choline diet or a high TMAO diet [54]. Compared to the control groups, DMB treatment regulated the diet-induced adverse vascular remodeling by attenuating the flow-induced atherosclerotic lesion formation and the expression of NLRP3 inflammasome, the endoplasmic reticulum stress burden, and reactive oxygen species formation [54]. Roberts et al. assessed the importance of a crucial microbial TMA-generating enzyme pair, CutC/CutD [101]. In a well-designed study, they proposed multiple halomethylcholines with the ability to prevent platelet activation and thrombus formation in vitro, without noting an increased bleeding risk [101]. Among the mentioned molecules, iodomethylcholine and fluoromethylcholine, which inhibit TMA-lyase activity similarly to DBM, have been assessed in other studies [55]. According to the histologic examinations, there was a reduction in the expression of inflammatory, fibrotic, and extracellular matrix remodeling markers in animals treated with iodomethylcholine. Apart from these effects, iodomethylcholine led to beneficial alterations in cholesterol and bile acid metabolism in wild-type C57BL/6J mice fed with a high cholesterol diet [102]. Fluoromethylcholine has been shown to reverse TMAO-induced tissue factor expression in a mouse model of arterial injury [56], indicating a possible anti-thrombotic role for this agent. Summarizing the findings mentioned above, inhibition of TMAO formation may have a role in the attenuation of atherosclerosis progression via reducing foam cell formation, inflammation, endoplasmic reticulum and oxidative stress, coagulation, and extracellular matrix remodeling (Figure 4).

Flavonoids, a large class of polyphenolic compounds, are present in tea, citrus fruit, citrus fruit juices, berries, red wine, apples, and legumes. In flavonoid aglycones, baicalein, fisetin, acacetin, and myricetin exhibited a decent binding effect with TMA-lyase, as did baicalin, naringin, and hesperidin in flavonoid glycosides [103]. Therefore, inhibition of TMA-lyase activity is another method of reducing TMAO concentration. This observation could partly explain the beneficial effects of flavonoids in CAD prevention [104]. Regarding other forms of diet, such as the Mediterranean diet, the findings are controversial, and sex-specific associations may be present [105,106]. Further research in this field is required to cement the role of specific dietary patterns in TMAO modulation.

To sum up, evidence in humans is all but lacking, and the use of TMAO inhibitors, or any other means of gut microbial modulation, is currently investigational. It is still too early to recommend microbiota measurements or modulation, by TMAO inhibitors or otherwise, for the specific purpose of preventing CAD.

#### 3.6.2. Fecal Transplantation

Fecal transplantation, a technique that has been of clinical use in the management of *Clostridioides difficile* infection [107], has also been evaluated concerning its potential cardiometabolic benefits in atherosclerosis and possibly CAD. An improvement in insulin sensitivity following the administration of gut microbiota from lean donors to men with metabolic syndrome has been reported, accompanied by an increase in the concentration of butyrate-producing intestinal bacteria (*Roseburia intestinalis*, *Eubacterium hallii*) [108]. A subsequent small scale, randomized controlled trial assigned 20 male patients with metabolic syndrome to vegan donor or autologous fecal transplantation [57]. Even though an alteration of host gut microbiota towards a vegan gut microbiota type was documented, this was not associated with reductions in TMAO or vascular inflammation through imaging or ex vivo peripheral blood monocyte production of pro-inflammatory cytokines [57]. These controversial human studies are accompanied by preclinical experiments. In a mouse model of autoimmune myocarditis, fecal microbiota transplantation promoted anti-inflammatory actions through restoration of the Firmicutes/Bacteroidetes ratio [109]. This is especially important, since a pro-inflammatory gut microbiome may be detrimental towards the development and progression of atherosclerosis [110]. In a recently reported experimental study involving the atherosclerosis-prone C1q/TNF-related protein 9-knockout mice, fecal microbiota transplantation from wild-type mice altered the composition of the host gut microbiota and abrogated the atherosclerotic lesions in the carotid artery following partial ligation [111].

## 4. Conclusions

There is currently an abundance of evidence that gut microbial dysbiosis is associated with CAD and cardiovascular risk. Low grade systemic inflammation induced by gut bacterial components or functions is probably the main underlying pathophysiological mechanism. Moreover, metabolites produced by the gut microbiota serve as markers of increased risk in cardiovascular patients, but causality in humans has not been proven yet. Numerous therapeutic interventions targeting the microbiota have been suggested and tried, with modest effect. Although some of the evidence is relatively strong, based on small randomized controlled trials and even meta-analyses, until larger randomized controlled trials are designed and conducted, manipulation of the gut microbiota in the context of CAD will remain far from clinical practice.

## Figures and Tables

**Table 1:** Selected preclinical and clinical studies of gut microbiome modulation demonstrating the effect of interventions in experimental, surrogate, or clinical markers of atherosclerosis.

Study	Year	Population	Intervention	Outcome
Probiotics				
Malik et al. [42]	2018	Male CAD patients	*Lactobacillus plantarum*	↓Inflammatory markers ↑Endothelial function No change in TMAO
Moludi et al. [43]	2021	CAD patients	*Lactobacillus rhamnosus*	↓Body weight ↓Inflammatory markers
Koppinger et al. [44]	2020	LDLr^−/−^ mice with ischemia/reperfusion injury	*Lactobacillus reuteri*	↓Infarct size
Sun et al. [45]	2022	CAD patients	*Bifidobacterium lactis* Probio-M8	Improvement in anginal, anxiety, and depressive symptoms ↓Inflammatory markers ↓TMAO ↓Proatherogenic aminoacids
Yoshida et al. [46]	2018	Female Wistar rats on a HFD	*Bacteroides vulgatus* *Bacteroides dorei*	Prevention of atherosclerotic plaque formation
O’Morain et al. [47]	2021	Male LDLr^−/−^ mice on a HFD	*Lactobacillus acidophilus* *Bifidobacterium bifidum* *Bifidobacterium animalis* subsp. *Lactis* *Lactobacillus plantarum*	↓Aortic root occlusion Atherosclerotic plaque stabilization ↓Inflammatory-, extracellular matrix remodeling-, and apoptosis-related gene expression
**Prebiotics**				
Aarsaether et al. [48]	2006	Patients scheduled for CABG	Β-1,3/1,6 glucan	↓CK-MB and cTnT
Merino-Aguilar et al. [49]	2014	Obese male Wistar rats	Fructooligosaccharides	↓Body weight ↓Inflammatory markers Improved lipid profile
Dehghan et al. [50]	2016	Female patients with DM	Oligofructose-enriched inulin	↓Inflammatory markers ↓Blood pressure Improved lipid profile
Parnell et al. [51]	2009	Overweight patients	Oligofructose	↓Body weight
**Synbiotics**				
Tajabadi-Ebrahimi et al. [52]	2017	Diabetic patients with CAD	*Lactobacillus acidophilus* 2 × 10^9^ CFU/g *Lactobacillus casei* 2 × 10^9^ CFU/g *Bifidobacterium bifidum* 2 × 10^9^ CFU/g 800 mg inulin	Improved insulin-glucose homeostasis Improved lipid profile
**TMAO Inhibitors**				
Wang et al. [53]	2015	ApoE^−/−^ mice	DMB	↓Foam cell formation ↓Atherosclerotic lesion development
Chen et al. [54]	2022	Wild type mice with partial carotid artery ligation	DMB	↓Vascular remodeling ↓NLRP3 inflammasome expression ↓Endoplasmic reticulum stress ↓Reactive oxygen species formation
Organ et al. [55]	2020	Wild type mice with transient aortic constriction	Iodomethylcholine	↓Adverse cardiac remodeling ↓Inflammation, fibrosis, and extracellular matrix remodeling
Witkowski et al. [56]	2021	Mouse model of arterial injury	Fluoromethylcholine	↓Tissue factor expression
**Fecal Transplantation**				
Smits et al. [57]	2018	Male patients with MetSy	Fecal transplantation from vegan donors	No alterations in TMAO or vascular inflammation

CAD: coronary artery disease, TMAO: trimethylamine N-oxide, LDL: low-density lipoprotein, HFD: high-fat diet, CABG: coronary artery bypass grafting, CK-MB: creatine kinase-myocardial bound, cTnT: cardiac troponin T, DM: diabetes mellitus, CFU: colony-forming unit, DMB: 3,3-dimethyl-1-butanol, MetSy: metabolic syndrome, ↓: decreased, ↑ increased.
